# Author response to ‘Comparing analgesic effect of regional block after hip arthroscopy’

**DOI:** 10.1093/jhps/hnae007

**Published:** 2024-05-16

**Authors:** Liangjing Yuan, Chengshi Xu, Ye Zhang, Geng Wang

**Affiliations:** Department of Anesthesiology, Beijing Jishuitan Hospital, Beijing, 100000, China; Department of Anesthesiology, Beijing Jishuitan Hospital, Beijing, 100000, China; Department of Anesthesiology, Beijing Jishuitan Hospital, Beijing, 100000, China; Department of Anesthesiology, Beijing Jishuitan Hospital, Beijing, 100000, China

To the Editor:

Our team has responded comprehensively to some questions regarding this article as follows:

(i) Regarding the preoperative pain situation of patients, a surgeon has evaluated it before admission. Patients with severe pain will choose other treatment methods, so the pain level of enrolled patients is similar.

(ii) Regarding the evaluation of muscle strength after 30 min of block, previous studies have only used the research method described in this article to evaluate muscle strength. The inclusion criteria before selecting cases also include the absence of movement disorders in the lower limbs. Therefore, we have a reason to believe that the evaluation of exercise is reasonable.

(iii) Regarding effective compression, this point was also considered by the research team at the beginning of the design, but due to the fact that not all pain relief pumps in the research center have this function, in order to maintain consistency, this item has not been statistically analyzed.

(iv) We have recorded and compared patient satisfaction. However, the relevance to the main point of this article is relatively small, and we will express it in the sequential research article.

Thank you for your attention. We hope that our reply can dispel your doubts.

